# Healthcare inequities and barriers to access for homeless individuals: a qualitative study in Barcelona (Spain)

**DOI:** 10.1186/s12939-021-01409-2

**Published:** 2021-03-20

**Authors:** Andrés Cernadas, Ángela Fernández

**Affiliations:** 1 Department of Political Science and Administration, Faculty of Political Science, Universidad de Santiago de Compostela, Campus Vida, S/N, 15782 Santiago de Compostela, Spain; 2 Graduate in Political Science and Master in Equality, Gender and Education, Universidad de Santiago de Compostela, Santiago de Compostela, Spain

**Keywords:** Healthcare inequality, Homeless people, Qualitative research, Barriers to access

## Abstract

**Background:**

In Spain, homeless individuals have lower perceived quality of health than the rest of the population and their life expectancy is 30 years lower than the national average. While the Spanish health system provides universal access and coverage, homeless individuals do not access or use public care enough to maintain their health. The objective of this study is to determine if homeless individuals can access public health services in conditions of equality with the rest of the population, as established in healthcare legislation, and to better understand the causes of observed inequalities or inequities of access.

**Methods:**

A detailed qualitative study was carried out in the city of Barcelona (Spain) from October 2019 to February 2020. A total of nine open and in-depth interviews were done with homeless individuals along with seven semi-structured interviews with key informants and two focus groups. One group was composed of eight individuals who were living on the street at the time and the other consisted of eight individuals working in healthcare and social assistance.

**Results:**

The participants indicated that homeless individuals tend to only access healthcare services when they are seriously ill or have suffered some kind of injury. Once there, they tend to encounter significant barriers that might be 1) administrative; 2) personal, based on belief that that will be poorly attended, discriminated against, or unable to afford treatment; or 3) medical-professional, when health professionals, who understand the lifestyle of this population and their low follow-through with treatments, tend towards minimalist interventions that lack the dedication they would apply to other groups of patients.

**Conclusions:**

The conclusions derived from this study convey the infrequent use of health services by homeless individuals for reasons attributable to the population itself, to healthcare workers and to the entire healthcare system.

Accordingly, to reduce inequities of access to these services, recommendations to healthcare service providers include adapting facilities to provide more adequate care for this population; increasing sensitivity/awareness among healthcare workers; developing in situ care systems in places where the homeless population is most concentrated; and establishing healthcare collaboration agreements with entities that work with this population.

## Background

After access to food, healthcare stands out as the primary service for maintaining and improving the well-being of a society. Countries shape their public health systems according to societal models that correspond to values shared by most of the citizens in a specific territory. By studying health policies, it is possible to analyze the sensitivity of a given political system to the population as a whole and especially to disadvantaged collectives. Such research also provides insight into how measures adapt to the particularities of the most vulnerable people in a society, to guarantee access to public health services in conditions of effective equality [1, 2].

In Spain, the General Law on Healthcare of 25 April 1986 (LGS, *Ley General de Sanidad*) was published with the political intent of making effective the right to health protection, based on Article 43 of the Spanish Constitution and other concordant norms. Two fundamental precepts can be highlighted from this law, which lays the foundations for public health care in the country. The first concerns coverage and establishes that “all Spaniards and foreign citizens residing in the national territory have the right to health protection and medical attention” (Art 1.2), Second, the law establishes that access to health services is granted under conditions of effective equality, based on the maxim of “equal access for equal need”*,* and that healthcare policy will be oriented toward overcoming social imbalances (Art. 3.2 and 3.3).

Though the LGS establishes the principle of universal access, significant inequalities and inequities that affect vulnerable populations can be detected in practice, as in many other countries [3–8]. Numerous studies indicate a connection between social inequalities and health inequities [9–13], while also suggesting that health inequalities stemming from social inequalities may be increasing [14–16]. This evidence has led different organizations such as the World Health Organization (WHO) or the United Nations (UN) to take up this problem and incorporate it into their main goals and objectives for the future.

### Inequalities and inequities

In this study “health inequalities” refer to differences, variations and disparities in the health of individuals or population groups, without imposing any moral, ethical or value judgements on the observed differences [17]. In other words, it refers to any quantifiable aspect of health that varies among individuals or socially relevant groups [18]. Meanwhile, “health inequities” refer to avoidable inequalities that are considered unjust or that emanate from some form of injustice, based on the governing concept of ‘justice’ in each society [19].

In this sense, it could be said that inequities are differences in health that might be avoided through adequate means [20]. Therefore, states that have historically made it a fundamental objective to guarantee universal access to healthcare and establish a social security system [21] for the entire population should bear the responsibility for reducing the inequalities and inequities that affect the most vulnerable populations. Through public policy and specific policies targeting vulnerable collectives [22, 23], these states must assume the challenge of correcting and minimizing healthcare inequities by eliminating barriers to access [24] or the implementation of positive discrimination measures.

Homeless individuals have no political representation to advocate their interests, which places them in a severe situation of marginalization and institutional invisibility [25]. The absence of political and social representation limits them when it comes to advancing their demands and requiring that their legal rights –those recognized by the legal system– be upheld.

In this context, the study presented here explores equity of access and use of public health services by the homeless population in the city of Barcelona. It seeks answers to the following questions: (i) Are homeless individuals accessing public health services in conditions of equality, as established in Spanish laws on healthcare?; (ii) If homeless individuals are not accessing public health services in conditions of equality with the rest of the population, what are the causes, barriers to access and consequences for the affected population?; and (iii) What recommendations or proposals can be made for practitioners and policymakers to reduce healthcare inequities for this population?

## Methods and participants

### Study design

To determine how homeless individuals perceive and describe their overall health status and their experiences of accessing public healthcare services, a qualitative study was designed based on in-depth or semi-structured interviews and focus groups. The design accommodated the usual requirements of information saturation and triangulation of sources, observers and methods.

We did nine open, in-depth interviews of homeless individuals on the streets or in a social center, along with seven semi-structured interviews with key informants (doctors, social workers and primary/urgent care nursing staff) at their worksites. All the interviews were audio recorded and fully transcribed. Two people from the work team were present at all interviews. The first set of interviews lasted from 90 to 130 min (average 126 min) and the second from 30 to 45 min (average 41 min).

Two focus groups were also created. One was composed of eight individuals who at that time were living on the streets. The group was supervised by the researchers. The other group consisted of seven individuals working in healthcare and social services (Table 1). In that group, the ideas from various professional spheres could be nuanced, discussed and contrasted in a way that complemented the more compartmentalized information from the interviews. Information saturation was achieved when the last two interviews did not provide any new or additional information.

**Table 1:** Profile of healthcare professionals who participated in the interviews

Code	Role	Sector
P.18	Social worker	Hospital social work
P.19	Social worker	NGO social work
P.20	Doctor	Family medicine
P.21	Nurse	Primary care
P.22	Doctor	Family medicine
P.23	Nurse	Urgent care
P.24	Social worker	Barcelona municipal government

For source triangulation, information was gathered from the homeless population, hospital primary/urgent care workers, and key informants from social entities that serve this population. The work team included the authors of this study and two collaborators with experience in these research techniques. They assisted with data collection and observation triangulation during the first phase of analysis. The method involved close-up observation and detailed analysis of the interview transcripts by the four members of the work team. Each team member developed their own conclusions from the information available about the homeless population and the general population, using a comparative method of contrasting known reality with observed reality [26].

There were no significant discrepancies among observers; any that did arise were resolved on the basis of higher incidence in the information sources used. Precedence was given to conclusions supported by more information sources.

No specific codification technique was applied. The participant responses were grouped by categories (see Results) corresponding to the main objectives of the study [27].

The results were analyzed from an inductive focus, which seemed the most appropriate method. Various authors have applied it to similar qualitative research [28–31].

### Location

The study was done in the city of Barcelona (Spain) from October 2019 to February 2020.

Barcelona is the second-largest city in Spain, both in population and economic activity, and rivals Madrid as the city with most homeless people. Its location in the Mediterranean basin and its mild weather make it attractive for homeless individuals from colder areas inside or outside Spain. According to the report *Quien duerme en la calle (Who Sleeps in the Street?)* by the Barcelona Municipal Government, in September 2018 there were 2452 homeless individuals residing in the city, about a thousand of whom were sleeping on the streets every day [32].

### Sample and participants

#### Homeless individuals

The homeless individuals who participated in the interviews and the focus group were approached on the street and asked to participate voluntarily, being assured of the strictest anonymity. Along with the general criterion of having been living on the streets for at least six months at the time of the interview, gender was considered so that men and women would be represented in the study. Age range was also taken into account. One in three of those approached participated in the study. Six of the interviews took place in parks, plazas and gardens; the other three in social centers.

The completely open and in-depth interviews explored diverse aspects related to the participants’ health and use of healthcare services (see Table 2).

**Table 2:** Profile of the homeless individuals interviewed

Code	Role	Age	Sex	Place of origin
P.1	User	51 years	Male	Alicante
P.2	User	57 years	Male	Malaga
P.3	User	63 years	Male	Girona
P.4	User	54 years	Male	Guadalajara
P.5	User	58 years	Male	Lugo
P.6	User	49 years	Male	Barcelona
P.7	User	64 years	Female	Bilbao
P.8	User	51 years	Male	Cordoba
P.9	User	47 years	Female	Valladolid

The focus group participants –not the same people as those interviewed individually– were recruited using the same method, except that two of them were contacted through a reference NGO (Non-Governmental Organizations), in this field (Table 3).

**Table 3:** Homeless individuals focus group composition

Code	Role	Age	Sex	Place of origin
P.10	User	56 years	Male	Pamplona
P.11	User	56 years	Male	Cadiz
P.12	User	50 years	Male	Oviedo
P.13	User	50 years	Male	Tarragona
P.14	User	66 years	Male	Castellon
P.15	User	53 years	Female	Barcelona
P.16	User	55 years	Male	Segovia
P.17	User	52 years	Male	Zaragoza

The meeting was coordinated and supervised by two members of the work team, audio recorded and transcribed completely. It lasted 141 min and took place on the premises of a foundation not related to the population under study.

#### Health professionals

Health professionals were recruited from primary care centers in municipal districts with higher numbers of resident homeless individuals, according to data from previous city censuses. The proposal was sent to the director of the healthcare center, who communicated it to medical and nursing staff. Those who agreed were interviewed. The interviews were done at their workplace and the participants were assured of the strictest anonymity.

#### Key informants

The key informants participated in the interviews and the focus groups. They were recruited at care centers in the city districts with the highest numbers of homeless individuals. We looked for people with ample experience attending to the homeless population and who belonged to institutions that habitually worked with such groups.

#### Focus group of professionals and key informants

The focus group of health professionals and key informants consisted of people who worked daily with this population, attending to their health and social care needs. It was a mixed group, so that the opinions from the diverse participants and sectors could be expressed and nuanced (Table 4).

**Table 4:** Profile of professionals who participated in the discussion groups

Code	Role	Sector
P.25	Hospital Social Worker	Catalan Institute of Health (ICS), Primary Care
P.26	Hospital Social Worker	Catalan Institute of Health (ICS), Hospital
P.27	Municipal Social Worker	City of Barcelona
P.28	Nurse	Emergency care
P.29	Nurse	Primary care
P.30	Doctor	Primary care
P.31	Doctor	Primary care
P.32	Community Social Worker	Comunitat Sant Egidi social project

Note: At the end of the interviews and the focus groups, the homeless individuals who participated in the study were compensated with a small amount of money that was never mentioned beforehand

We chose not to remunerate the professionals who were interviewed at their workplaces, on the understanding that their collaboration could be considered part of their work

However, it seemed appropriate to remunerate the professionals who participated in the discussion groups with a small gift to compensate them for their time – over two hours of discussion – and transit efforts

The meeting was coordinated and supervised by two members of the work group, audio recorded and completely transcribed. The meeting lasted 126 min and took place on the premises of a foundation not related to the population under study.

The information gathered from the interviews of the members of the collectives participating in the study was analyzed first, to identify the most relevant aspects for this study. After that, the information obtained from the key or privileged informants was added and the most relevant aspects were reformulated, then later contrasted and refined in the focus groups.

All the information gathered in the interviews and focus groups was transcribed. The information was then sorted and systematized by hand, because digital processing would not capture all the relevant nuances.

## Results

With the study objectives to guide us, after compiling the information, it seemed useful to organize the findings into two tables that contain the responses of the participants in the field, which were originally transcribed verbatim in the language of the speaker (Spanish, Catalan). Table 5 contains the translated responses of healthcare and social workers, while Table 6 contains the responses of homeless individuals.

**Table 5:** Sample responses from healthcare professionals

Barrier		Responses
**Personal resources**	**Cognitive capacity, healthcare culture and understanding of the system**	(1) P.18 “Some people have a medical card and some don’t; normally they don’t have an assigned doctor and they have to be registered as city residents and fill out a series of papers, something they understand as us not wanting to attend to them. This is an important barrier.” (2) P.19 “For homeless people everything is too bureaucratic, so they get the idea that no one wants to help them in the health centers, and they leave.” (3) P.24 “Many of them have no documents, because their documents have been stolen, or lost, and they never replaced them. Many don’t care about it, because they prefer to die.” (4) P.20 “The fact that a homeless person ventures into a clinic or emergency room or has a medical card does not imply good access. I think access is deplorable.” (5) P.25 “Normally, these people tend to make occasional or extreme use of health services, they only go to emergency care and then there is no continuity, among other things.” (6) P.26 “The situation is that this population has no territorial links, so they don’t go to their local clinics for care, because they don’t know which one they have been assigned to.”
	**Mobility, personal autonomy and social assistance**	(7) P.20 “On the 061 Emergency line they are really arrogant. When I call them to come for a user who is in alcohol or drug crisis and cannot move, I get answers like, ‘and you’re calling us about *this*?’ (8) P.24 “My perception of the people who are in the street is that they don’t really care about their situation and logically the same thing happens on the healthcare side also, it’s not their priority and they don’t seek care until the symptoms of the illness they are suffering from become unbearable.” (9) P.25 “From the healthcare side it’s a situation where you try to give medical and pharmaceutical treatment… but the person also needs a bath, lodgings, etc. Health services can’t provide that, it’s a social services issue. Once there is minimal social processing, then they can see the doctor, but if the social side is not addressed, it’s impossible to do anything there.” (10) P.26 “Healthcare displaces social [issues], social displaces healthcare.” (11) P.22 “This population thinks that doctors represent a society that has rejected them and which they do not trust, so the solution is to go to them [the homeless], not to wait for them to come.”
**Adaptation of the healthcare system and lack of resources**		(12) P.27 “The main problem? There are no resources designated to this population.” (13) P.28 “The doctors are very aware that homeless people do not follow the treatments prescribed to them and so have the sensation that they are wasting their time on them. This may be why they don’t pay much attention to them.” (14) P.21 “The case of a man with respiratory problems. Hostels and shelters don’t allow oxygen cylinders and these people go from one to another.” (15) P.22 “As professionals we are not sufficiently prepared, nor do we have the resources, to attend to this population.” (16) P.21 “It is heroic when homeless people manage to see a doctor, they have had to overcome numerous obstacles.” (17) P.28 “The health system is very rigid about access for these people, it’s difficult for them to understand all this bureaucracy.” (18) P.27 “What happens is that homeless individuals do not easily access the healthcare system, precisely because of how they live.” (19) P.29 “A homeless person comes to our center and the first thing they encounter is an admin person who doesn’t stop asking questions: are you a registered resident? Do you have an assigned doctor? Do you have a medical card? …” (20) P.20 “The situation sometimes depends a lot on the good will of the medical worker. Me, for example, when someone like this comes in, I ask that they be assigned to me, because otherwise, since they have no address, no one can be assigned to them.” (21) P.31 “There are a lot of administrative hang-ups that slow down care, then when you finally manage to see the person and give them the immediate treatment they need and get them an appointment with the social worker, the person has already left, without medication, without the smallest chance of follow-up.” (22) P.29 “The system has been reducing the obstacles to access and today it is practically universal, but even so, there are some populations with special characteristics who do not come in, you have to go find them. And in this group, we find the homeless.*”* (23) P.18 “There need to be units that go to them. The role of the street educators is essential.” (24) P.30 “Often time is the issue: we have seven minutes to address medical problems, social problems, relational and communication problems… You feel powerless, you see the situation is unresolvable and no matter what you do it’s not worth the effort and they won’t follow your instructions, because they can’t.” (25) P.28 “I believe the problem is that there is no coordination, no integrated effort that covers everything. The problem is not the nursing staff, the doctors, or the social workers; the problem is [lack of] coordination.” (26) P. 30 “Coordination is shoddy because there are no standards for this population and they always end up looking to see who is on call, to see if they can send them the cases or not”.
**The role of healthcare professionals**	**External appearance of the user and attitudes of professionals**	(27) P.32 “The main problem for the homeless population in accessing health care is that it is heavily influenced by their outward appearance. Both users and professionals are aware that their outward appearance has a lot to do with access conditions and standard treatment.” (28) P.31 “You encounter a man who smells terrible sitting in the waiting room, at a moment when you have forty visits and five minutes for each one; the people waiting begin to grumble that the man is bothersome… then, after a while, you get him into the room and you find a person who is difficult to understand, whom you know nothing about because it’s the first visit. Then, when you say ‘undress’, you have to make a massive effort at professional discipline to get close and not vomit.” (29) P.27 “There’s a whole range of prejudices on the part of the medical professional that make it so that the homeless patient always ends up getting worse treatment: because they drink alcohol, because they have mental problems, because of their drug addictions, because of their physical appearance… In these cases, the professional examines them from a meter away. Dirtiness is always a factor in care discrimination, in fact, there are auxiliary staff who wash them up so that the doctor will treat them like anyone else and not be discriminatory.” (30) P.26 “Sometimes they are even treated first just so that their physical appearance and smell do not bother the other patients who are waiting.” (31) P.21 “Sometimes people arrive lice-ridden, filthy, drunk, malnourished… For special cases, there are two closed examination cubicles and they are left there to wait, away from the others.”
	**Information provided by the system and medical professionals**	(32) P.25 “Homeless people are checked, but the doctors don´t speak to them, they always speak to the person accompanying them.” (33) P.19 “Sometimes homeless people find that they have seen many different doctors who have told them completely different and contradictory things in a very short time.” (34) P.30 “I think that apart from all the problems with social services and difficulties with networking care, there is a part that we (healthcare workers) should try to facilitate.” (35) P.32 “Doctors are not concerned with the personal situation of the users, they attend to them, admit them to hospital if necessary, and the rest are problems for the administrative staff.”
**Attitudes and habits related to health**	**Following protocols and continuity of care**	(36) P.29 “The problem is, how can you ask a street person to follow a prescribed treatment, when they live day to day? People living on the streets can’t care for themselves.” (37) P.19 “Sometimes they have so many problems… not just a housing problem. Sometimes they are homeless, but also illegal residents, they have mental health issues, they are alcoholics or addicts… Then you look at them and you think ‘where do I start?’” (38) P.24 “I think that subconsciously, medical professionals avoid the homeless a bit. They [health workers] know their habits and that they won’t follow treatments, so they [the homeless] are relegated and end up being treated differently”. (39) P.31 “We have to deal with the real situation that we have: taking a person’s blood pressure, when you know there will be no follow-up if you treat them for hypertension, is a waste of time.” (40) P.32 “With the homeless, the idea is: *‘whatever they have, don’t put yourself out, there’s no solution and in the end we all have to die’.* They are relegated in treatments, they wait longer, because they don’t complain and ‘*we’re doing them a favor*’ by seeing them at all.” (41) P.30 “There is no follow-up, it’s really difficult.” (42) P.23 “The argument goes like this: what’s the point of solving their health problem, if no one can ensure that they will follow the prescribed treatment? The result is that they get patchwork answers rather than solutions for their real problems.” (43) P.18 “Without a medical card, we sent them to *Fundación Trueta*. The problem is that this population often present mental health problems and that entity does not provide medications for them. (44) P.27 “They have difficulties in accessing primary care and therefore they also don’t have access to a more ongoing treatment path”. (45) P.23 “Another problem is the follow-up for these people. Because any person, when you finish treating them in an emergency service, you say, ‘all done, now go home and finish recovering’, but these people don’t have a home to recover in. And I don’t think this is due to the poor functioning of healthcare, but because this population are not taken into account.”

**Table 6:** Sample of user opinions

Barrier	Responses	
**Personal resources**	**Economic capacity**	(1) P.2 “I don’t earn anything, not a cent. But I don’t care, I don’t eat much, I don’t care if they don’t give me anything”. (2) P.11 “A lot of the medicines we take aren’t covered by social security [medical card].” (3) P.5 “I don’t get paid any money, the social worker told me that with a few papers I could get that *PIRMI*^1^, but I don’t want anything, I go round the street cafés and ask for some cigarettes and that’s it.” (4) P.6 “I don’t eat much, but sometimes a little grandmother brings me food.” (5) P.10 “I go to the Red Cross and there they give me medicines when I need them, I can’t pay for them.”
	**Cognitive capacity, healthcare culture and understanding of the sysm**	(6) P.1 “A *morito* (…) stole my wallet and [medical] card (…).” (7) P.4 “Yes, I have a medical card, but I’m not going back to the doctor, what I want is to die.” (8) P.17 “I have a medical card, but I don’t go to the doctor. And when I see that I’m going to die, two days before I’ll throw my ID card in the trash, I don’t want them to know who died, nobody cares. They ignore me? Fine, I don’t need anything.”
	**Mobility, personal autonomy and social support**	(9) P.5 “I was lying in the street and I couldn’t even move and the ambulance [workers] didn’t want to pick me up. Yet later, later in the hospital they were very good to me, but in the ambulance it was as bad as it gets.” (10) P.1 “Once they had to call the police because the ambulance didn’t want to come for me. And with the police there and everything they didn’t want to take me. Why is that?” (11) P.4 “Look at me, in a wheelchair, I can’t move … and when I go to ask for something [public assistance] they ask me for a work contract. Do you think they’ll give me a work contract in my state?” (12) P.14 What do you mean am I feeling ok? Don’t you see me? Out here, in the cold? I sleep here, on this bench, every day, I always sleep here, you can always find me here.”
**Adaptation and lack of resources in the healthcare system**		(13) P.10 “I have no complaints about *Seguridad Social* [state health care], we just have to adapt to the system.” (14) P.4 “In the hospital they send us street people from one place to another, no one wants to help us, they don’t want to work or bother with us.”
**The role of healthcare professionals**	**External appearance of the user and attitudes of healthcare professionals**	(15) P.6 “When I go to the doctor, I always go cleaned up and they always treat me well, but other people, I don’t know, people complain about it.” (16) P.14 “They treat you bad, they smell [alcohol on] my breath and send me back out the door to the street.” (17) P.15 “I went to pick up an x-ray and the first thing the doctor had written on the report: ‘alcoholic’.” (18) P.9 “When you go to the doctor, as a general rule, you say that it hurts here or it hurts there, and the first thing they say is: ‘do you drink? Then it’s your liver’.” (19) P.7 “As soon as I entered the hospital, they told me that they’d see me after I’ve sobered up.” (20) P.2 “They treat you bad, they kick you out as soon as they see you because they know you have drinking problems. And then you think, ‘why go to the doctor if I’m going to keep on drinking?’”
	**Information provided by the system and professionals**	(21) P.8 “If the doctor explains things to me, I understand them, but they don’t explain anything to me.”
**Attitudes and habits related to health**	**Health status, perceived health and use of public health services**	(22) P.12 “I broke this finger here, but it will heal, I never go to the doctor, never, no doctor. If I get really bad one day and they take me to the hospital… fine, but I never go to the doctor. Why would I? What I want is to die.” (23) P.9 I have been really ill, with high fever, and that was when I went to the doctor. But I waited until the last moment. Not because of laziness, but so I wouldn’t have to stop drinking. And then you go to the doctor and you’re not going to tell them ‘look, I just like to get drunk’. It’s embarrassing, it’s hard to do.” (24) P.3 “Me, as long as I can manage on my own… If one day I have an attack or something, well, I’ll have to go to the doctor, but for now…” (25) P.11 “I never go to the doctor, an ambulance comes at the last moment if someone from the neighborhood who knows me sees me and calls, when I can’t stand up, but I never go and never call the ambulance.” (26) P.17 “I don’t want to care for myself, what for? What I want is to die, what can I do? Nothing, I’m good for nothing, what I want is to die.” (27) P.13 “If the ambulance doesn’t come for me I won’t go to the hospital on my own, even if I’m really bad off.” (28) P.2 “To go to the doctor I have to be really sick. It’s true that sometimes doctors come around here, but I don’t go myself, if I die, they’ll just have to bury me.” (29) P.9 “I don’t want to know anything about doctors, the only thing that has held up at this point is my health, I don’t have any problems.”
	**Following protocols and continuity of care**	(30) P.13 “I’m diabetic, but I don’t take the pills anymore, they cost almost 5 euro and I don’t have money. My legs and arms swell up with the diabetes, but what can I do? (31) P.14 “You don’t follow the treatments. You take [the medicine for] two or three days, mixed with booze… or you don’t.” (32) P.3 “I see poorly, because I haven’t changed my lenses for 8 or 10 years, I don’t have money.” (33) P.8 “There’s no problem, you can get the medicines anywhere, there are some nuns that I go to see and they give me the medicines I need.” (34) P.15 “On the streets, you don’t take your medications, I speak for myself and for the others, no one takes them.” (35) P.12 “We’re people who are here today and there tomorrow, we move around constantly and it’s a hassle to carry your medicine around. Call it laziness, or lack of interest, but you don’t take it and you don’t follow through on the therapies like you should.” (36) P.16 “If the medications are not covered by state insurance and I can’t pay for them, they don’t get taken.”
**Perceived quality of care**	(37) P.17 “I have a lot of problems because a security guard beat me in the hospital. And with the doctors, it goes in one ear and out the other.” (38) P.16 “They stabbed me three times, it was nighttime and they took me to hospital. And at 1 in the morning, when they had finished treating me, they can’t think of anything better to do, on a cold night like tonight, than ‘out you go, back on the street with you’. Raining, with no clothes and nothing. Then I had terrible anemia, I was in hospital for six days, and they were very decent, as good as it gets, I had everything I needed.” (39) P.7 The doctor who saw me is one of the worst I’ve had in my life, had no manners, no shame, no humanity. The first thing they should have is humanity. I don’t want to go back to the doctor again, I want to die as soon as possible.” (40) P.1 “I was bleeding from the nose and mouth, and the only thing he could say to me, this man from the hospital at three in the morning, was that I had go back out on the street. I asked if I could talk to someone, because I have nowhere to sleep and with all this I get dizzy and I might die and they’d have to pick up my cadaver. And they said I couldn’t stay, because there were no beds. At least let me stay in the waiting room until morning and then I can go myself!”	

^1^The *PIRMI* or Renta Mínima de Inserción (RMI) is a regional minimum insertion wage offered by the Generalitat de Cataluña to individuals or families without resources for subsistence or rights to other economic assistance

The information gathered on barriers and difficulties of access are arranged into three categories: 1) barriers attributable to individual capacities or incapacities among members of the population itself; 2) barriers attributable to the health system and ways of organizing service that do not adapt to the particularities of homeless individuals or offer specific programs oriented to that population; and 3) barriers attributable to healthcare workers, who with their work dynamics, biases and behaviors can significantly increase inequity of service to a population that is already especially sensitive.

The findings manifest the existence of a series of limitations that impede access of homeless individuals to healthcare services in conditions equal to those of the general population. Therefore, their possibilities of using the services offered by the healthcare system work better in theory than in reality. Legally, access should not be a problem, but during the field work the users revealed – and the other informants corroborated – that in practice these people encounter diverse barriers. The main barriers are described below.

### Personal resources

#### Economic capacity

Scarcity of resources is very severe in this population, which translates into a series of deficits that impede homeless people from regularly attending to their needs for food, shelter, hygiene or personal care. The following are some of the responses of homeless individuals who were asked about the daily limitations derived from their lack of economic capacity:

*“I don’t earn anything, not a cent. But I don’t care, I don’t eat much, I don’t care if they don’t give me anything”* [P.2, user].

*“I don’t get paid any money, the social worker told me that with a few papers I could get that PIRMI, but I don’t want anything, I go round the street cafés and ask for some cigarettes and that’s it”* [P.5, user].

This also makes it difficult for them to access medication and pharmaceuticals not covered by the state system. Lack of access has serious repercussions on their health, as they cannot pay for necessities such as glasses, dental and orthopedic prosthetics, etc., and makes it impossible for them to maintain adequate levels of functioning and autonomy. When asked about access to specific medications or prosthetics, the homeless participants confirmed that high pharmaceutical costs combined with their limited economic resources meant that they often could not access the treatments and prosthetics they needed, or that they did so only with the help of entities working to assist this population:

*“I’m diabetic, but I don’t take the pills anymore, they cost almost 5 euro and I don’t have money. My legs and arms swell up with the diabetes, but what can I do”* [P.13, user].

*“I see poorly, because I haven’t changed my lenses for 8 or 10 years, I don’t have money”* [P.3, user].

#### Knowledge levels, healthcare culture and understanding of the system

Habitually, the homeless population presents an important lack of understanding concerning their rights, those of the organization and how the healthcare system functions in general. They often do not know which local primary care center they are assigned to or what level of healthcare they should go to for a given medical condition. One of the social workers who participated in the discussion group attributed it to the fact that this population is not linked to any specific healthcare center:

*“The situation is that this population has no territorial links, so they don’t go to their local clinics for care, because they don’t know which one they have been assigned to”* [P.26, social worker].

Homeless individuals often have no medical card (TSI) because it has been stolen, ruined, or lost; they may have a provisional card or perhaps they never applied for one. Precarious housing aggravates the situation. Without a medical card, homeless individuals are unlikely to have a family doctor assigned to them, so they go to emergency care. As a result, they have no clinical history, no pharmaceutical coverage and no access to preventative treatments or follow-up care. Throughout the study, both professionals and homeless individuals referred to this situation in interviews and focus groups:

*“Some people have a medical card and some don’t; normally they don’t have an assigned doctor and they have to be registered as city residents and fill out a series of papers, something they understand as us not wanting to attend to them. This is an important barrier”* [P.18, social worker].

*“Many of them have no documents because their documents have been stolen, or lost, and they never replaced them. Many don’t care about it, because they prefer to die*” [P.24, social worker].

However, even when homeless individuals have a health ID card, the problems persist and are associated with other aspects. In their responses, some patients even expressed significant disinterest in caring for their health:

*“I don’t want to care for myself, what for? What I want is to die, what can I do? Nothing, I’m good for nothing, what I want is to die”* [P.17, user].

*“To go to the doctor I have to be really sick. It’s true that sometimes doctors come around here, but I don’t go myself, if I die, they’ll just have to bury me”* [P.2, user].

However, these responses could be motivated to some degree by unsatisfactory prior experiences or lifestyle-based stigmas they might face when they go to a medical center, rather than mere disinterest in caring for their health.

Other times, problems with access may stem from bureaucracy and the formal or informal complexities that this population encounters in the public health system:

*“For homeless people everything is too bureaucratic, so they get the idea that no one wants to help them in the health centers, and they leave”* [P.19, social worker].

*“A homeless person comes to our center and the first thing they encounter is an admin person who doesn’t stop asking questions: are you a registered resident? Do you have an assigned doctor? Do you have a medical card? … ”* [P.29, nurse].

At other times, problems may be due to preconceived biases among the homeless population regarding the attention they will receive:

*“In the hospital they send us street people from one place to another, no one wants to help us, they don’t want to work or bother with us” [*P.4, user].

### Mobility, personal autonomy, and social support

As has been mentioned, homeless individuals tend to put off seeking care until the problem is very severe or do not go unless taken by healthcare workers. These have generally been called by another person and go to evaluate the situation on site, taking the person to a care center as needed. However, both the users and healthcare professionals who participated in the fieldwork described friction and even conflict between ambulance services and homeless individuals, stemming from poor or inadequate treatment and inadaptation of the service to the characteristics of the population:

*“On the 061 Emergency line they are really arrogant. When I call them to come for a user who is in alcohol or drug crisis and cannot move, I get answers like, ‘and you’re calling us about this??”* [P.20, doctor].

*“I was lying in the street and I couldn’t even move and the ambulance [workers] didn’t want to pick me up. Yet later, later in the hospital they were very good to me, but in the ambulance, it was as bad as it gets”* [P.5, user].

Homeless individuals have very reduced autonomy and lack the social support that in many cases is fundamental to successfully overcoming their illnesses. This was mentioned repeatedly by social workers and homeless individuals throughout the study:

*“From the healthcare side it’s a situation where you try to give medical and pharmaceutical treatment … but the person also needs a bath, lodgings, etc. Health services can’t provide that, it’s a social services issue. Once there is minimal social processing, then they can see the doctor, but if the social side is not addressed, it’s impossible to do anything there”* [P.25, social worker].

*“Look at me, in a wheelchair, I can’t move … and when I go to ask for something [public assistance] they ask me for a work contract. Do you think they’ll give me a work contract in my state?”* [P.4, user].

### Adaptation of the healthcare system and lack of resources

Added to the severe lack of resources that homeless people experience are the deficits of the healthcare system and social services, in terms of resources or lines of care aimed at covering the specific needs of this population. This also very negatively affects the health status of these individuals:

*“The main problem? There are no resources designated to this population”* [P.27, social worker].

*“As professionals we are not sufficiently prepared, nor do we have the resources, to attend to this population”* [P.22, doctor].

There is consensus among the various professionals that homeless individuals present greater difficulties in accessing public health services, because the system has no facilitating factors to correct these barriers:

*“The system has been reducing the obstacles to access and today it is practically universal, but even so, there are some populations with special characteristics who do not come in, you have to go find them. And in this group, we find the homeless”* [P.29, nurse].

*“Often time is the issue: we have seven minutes to address medical problems, social problems, relational and communication problems … You feel powerless, you see the situation is unresolvable and no matter what you do it’s not worth the effort and they won’t follow your instructions, because they can’t”* [P.30, doctor].

Homeless individuals are also aware that their appearance and way of life can predispose healthcare workers against them and sometimes acknowledge that they don’t do enough to adapt to the system:

*“I have no complaints about Seguridad Social [state health care], we just have to adapt to the system”* [P.10, user].

### The role of healthcare professionals

#### External appearance of the user and attitude of professionals

The interaction between the homeless population and healthcare professionals is highly conditioned by the outward appearance of the homeless individual. Their extreme physical presentation, often characterized by the impossibility of washing, the lack of routine personal care, or factors stemming from drug or alcohol addictions, greatly affects both the quantity and the quality of medical attention this population receives:

*“There’s a whole range of prejudices on the part of the medical professional that make it so that the homeless patient always ends up getting worse treatment: because they drink alcohol, because they have mental problems, because of their drug addictions, because of their physical appearance … In these cases, the professional examines them from a meter away. Dirtiness is always a factor in care discrimination, in fact, there are auxiliary staff who wash them up so that the doctor will treat them like anyone else and not be discriminatory”* [P.27, social worker].

*“The main problem for the homeless population in accessing health care is that it is heavily influenced by their outward appearance. Both users and professionals are aware that their outward appearance has a lot to do with access conditions and standard treatment”* [P.32, social worker].

Healthcare professionals concede that they are consciously or subconsciously influenced by the physical aspect of a homeless individual, which generates a dynamic of stigmatization and rejection:

*“You encounter a man who smells terrible sitting in the waiting room, at a moment when you have forty visits and five minutes for each one; the people waiting begin to grumble that the man is bothersome … then, after a while, you get him into the room and you find a person who is difficult to understand, whom you know nothing about because it’s the first visit. Then, when you say ‘undress’, you have to make a massive effort at professional discipline to get close and not vomit”* [P.31, doctor].

Homeless individuals also perceived this differential treatment and related it to their physical aspect, their living conditions or their addictions:

*“When I go to the doctor, I always go cleaned up and they always treat me well, but other people, I don’t know, people complain about it”.* [P.6, user].

*“When you go to the doctor, as a general rule, you say that it hurts here or it hurts there, and the first thing they say is: ‘do you drink? Then it’s your liver’”* [P.9, user].

#### Information provided by the healthcare system and professionals

The information generated by the system and provided to users by healthcare staff is not adapted to the circumstances of homeless individuals. Even when they receive the same information as other users, homeless individuals are less able to benefit from it for reasons ranging from disinterest –if unable to follow prescribed treatments, the information offered may be of little use to them– to basic comprehension to the attitude and sensitivity of the healthcare workers giving them the information.

Similarly, the information generated by the system –pamphlets, triptychs, brochures, legal information, informed consent, along with information related to prescribed medications and treatments– are not adapted to the homeless population.

Healthcare workers sometimes play a key role in critical situations by deciding not to interact directly with the homeless user, but with the person accompanying them, when there is one, or not interacting at all:

*“Homeless people are checked, but the doctors don’t speak to them, they always speak to the person accompanying them”* [P.25, social worker].

*“If the doctor explains things to me, I understand them, but they don’t explain anything to me”* [P.8, user].

This has very negative repercussions on the user, who remains uninformed, thinking the information was not meant for him or her, as it was not provided directly by the medical professional. Potentially more serious is the effect of this behavior on the homeless user, who may feel shunned by the professional, leading to greater disaffection with the system, less use and unattended needs.

### Health-related attitudes and habits

#### Health status, perceived health and use of public health services

Homeless individuals tend to be aware of their poor health. However, this is generally insufficient to motivate them to take the step and go to medical care, since their own health is not always their main priority. Often, they have nowhere to leave their belongings, so they put off visiting a medical center for as long as possible or simply do not go until their condition is critical or an ambulance comes for them. By then, their illness may have worsened:

*“I broke this finger here, but it will heal, I never go to the doctor, never, no doctor. If I get really bad one day and they take me to the hospital … fine, but I never go to the doctor. Why would I? What I want is to die”* [P.12, user].

*“Me, as long as I can manage on my own … If one day I have an attack or something, well, I’ll have to go to the doctor, but for now … ”* [P.3, user].

#### Follow-up protocols and continuity of care

Follow-up of certain medical treatments is often conditioned by the lack of economic resources that burdens this population. Many present chronic illnesses stemming from addictions, mental health problems or malnutrition. These require specific and ongoing treatments that are often excluded from public subsidy:

*“If the medications are not covered by state insurance and I can’t pay for them, they don’t get taken”* [P.16, user].

*“Without a medical card, we sent them to Fundación Trueta. The problem is that this population often present mental health problems and that entity does not provide medications for them”* [P.18, social worker].

Besides economic difficulties, the lifestyle of this population makes it extremely difficult for them to take medications regularly, at the recommended times and doses. As a result, their health deteriorates at a much higher rate than the general population:

*“You don’t follow the treatments. You take [the medicine for] two or three days, mixed with booze … or you don’t.”* [P.14, user].

*“Another problem is the follow-up for these people. Because any person, when you finish treating them in an emergency service, you say, ‘all done, now go home and finish recovering’, but these people don’t have a home to recover in. And I don’t think this is due to the poor functioning of healthcare, but because this population are not taken into account”* [P.23, nurse].

Being precariously housed and not having a medical card make continuity of care practically impossible. Without a medical card, there is no clinical history and no assigned family doctor. Continuity of care is thus extremely compromised by the inadaptation of the system to this population and the absence of resources and social support. These users find themselves in a situation of complete institutional neglect.

Accordingly, because they know or assume the lifestyle of these users, the healthcare professionals expressed that they end up doing minimal interventions, thinking that the person will probably not return or follow the prescribed treatment. This generates a sense that they are wasting their time:

*“With the homeless, the idea is: ‘whatever they have, don’t put yourself out, there’s no solution and in the end we all have to die’. They are relegated in treatments, they wait longer, because they don’t complain and ‘we’re doing them a favor’ by seeing them at all”* [P.32, social worker].

*“The doctors are very aware that homeless people do not follow the treatments prescribed to them and so have the sensation that they are wasting their time on them. This may be why they don’t pay much attention to them”* [P.28, nurse].

### Perceived quality of care

In many cases, the users who arrive at the care center feel that medical services have not been useful to solve their health problem, or that they have been treated less humanely due to their homeless condition:

*“The doctor who saw me is one of the worst I’ve had in my life, had no manners, no shame, no humanity. The first thing they should have is humanity. I don’t want to go back to the doctor again, I want to die as soon as possible”* [P.7, user].

Homeless individuals also perceive that the healthcare workers try to attend to their health problems but are insensitive to their vulnerable situation. Once the medical concern has been addressed, social concerns are dismissed:

*“They stabbed me three times, it was night, and they took me to hospital. And at 1 in the morning, when they had finished treating me, they can’t think of anything better to do, on a cold night like tonight, than ‘out you go, back on the street with you’. Raining, with no clothes and nothing. Then I had terrible anemia, I was in hospital for six days, and they were very decent, as good as it gets, I had everything I needed”* [P.16, user].

These bad experiences due to their appearance, the stigmatization of this population, the insensitivity of healthcare professionals, the conditions in which care is given or a combination of any of these can cause homeless users to become disaffected with the system and form a negative opinion regarding the quality of care in medical centers.

## Discussion

The objective of this study was to analyze inequities of access to public healthcare services by the homeless population residing in Barcelona, along with the causes of these inequities, using qualitative techniques such as interviews and focus groups.

The results of this study demonstrate that homeless individuals cannot access public healthcare services in conditions of equality, which constitutes an important inequity for this population group. The healthcare system in Spain is configured for a “standard patient” and does not easily adapt to those who present special characteristics. Homeless individuals encounter serious difficulties in making use of public healthcare services in conditions of equality. This generates important inequities in the system, which have been documented in the relevant literature [33].

One finding of the present study is that homeless patients often complained about the treatment they received from healthcare professionals. This can negatively affect future visits to healthcare centers, so it seems vital that a homeless individual’s first visit to a healthcare center, whether primary care or hospital emergency care, be a positive experience [34].

The target group also provided information about abandoning prescribed treatments due to the difficulties associated with follow-through in the absence of a home and adequate living conditions. This has been reported in other studies [35] and is something the patients associate with their way of life.

The present study corroborates recent research [36] in revealing that despite their diminished health –especially in pathologies such as chronic obstructive pulmonary disease, musculoskeletal disorders, tuberculosis, and skin and foot problems [37]– homeless individuals make less use of public assistance services and have lower life expectancy than the rest of the population. Sub-optimal use of healthcare services is related to various obstacles to access. These may stem from the homeless population itself, who may lack identification documents, maintain biases about the deficient attention they expect to receive, or indicate little regard for their own health. In other cases, barriers correspond mainly to lack of medical coverage in countries without universal access to healthcare services [38]. There may also be administrative and bureaucratic barriers related to contact personnel, or cultural and ethnic barriers derived from the behavior of healthcare workers [39].

One relevant result of this study has been to verify the persistence of aspects linked to the use of healthcare services, which for the patient are perceived as –and act as– important barriers to access that seriously affect equity of access and use of the system. These aspects are broadly supported by prior studies that have detected similar situations in other countries [40] and recognized by relevant professionals [41]. Along with barriers to access, the studies also refer to unsatisfactory use. This generates disaffection with the system among homeless individuals, who think they are not treated adequately, accorded sufficient respect or given sufficient attention [42]. Along the same lines, another recent study in Alabama (USA) reported de-humanized attention along with a lack of commitment and professionalism on the part of medical staff. This contributed to diminishing confidence in the medical system among homeless individuals [39, 43].

The present study verified that when homeless individuals approach a healthcare service, the first difficulty they encounter involves getting past contact personnel –security, administrative or reception staff– who act as gatekeepers, as other studies have also indicated [44]. Once these barriers are surmounted, homeless individuals must often also overcome the reluctance of medical professionals in order to receive attention under the same conditions as other patients. Their lifestyle, appearance and supposed addictions can negatively influence the willingness of these professionals.

Medical workers also know that these patients have low follow-through with treatments, often due to negligence, disinterest, or lack of economic resources to access medical supplies and equipment or medications. Thus, medical workers tend to do minimal interventions, as some relevant studies have highlighted [35].

Accordingly, homeless individuals may sometimes be stigmatized by healthcare workers. Within the medical profession, it is believed that this population sub-group does not adequately follow prescribed treatments or have personal habits that are compatible with what has been prescribed for their pathologies. As a result, this population may receive lower quality medical attention compared to others [45].

Sometimes, to avoid these situations and potential stigmatization by medical staff, the entities that work with homeless individuals accompany them to medical visits. Occasionally, they also provide a way for these individuals to wash and tidy themselves up, in hopes that they will be treated like standard patients rather than homeless patients by medical workers. However, while these entities may help the homeless patient to avoid stigmatization by healthcare workers, entity personnel may be creating stigma through the actions themselves.

Though there is ample consensus between the literature cited and some of the main findings of this study, the overlap diminishes when it comes to naming possible solutions for reducing the inequities that affect this population. Several studies based on means-testing programs [46] have demonstrated that attention to this population significantly improves when attention-specific, ad hoc programs are designed, as opposed to standardized care [47–50]. Along these lines, some studies address the most frequent pathologies among this population in an aggregate manner [51], while others analyse them separately to better assess them. These include nutrition [52], mental health [53, 54], and pathologies related to the consumption of alcohol [55] or the use of tobacco or other addictive substances [56, 57] to name a few. However, data from the present study make it possible to deduce that the problems of this population basically involve accessing standardized medical care. If equity in access and use of services could be assured, most of the problems would be solved.

In some places, healthcare programs have been implemented for homeless individuals that run parallel to the general system, such as medical interventions in homeless shelters in Amsterdam [58] and Leicester [59], or collaboration agreements with service sector entities in other places.

Given the attitude of this population, it would be easy to conclude that such programs would be ineffective, but experience indicates the opposite. Programs such as Housing First, which emerged in the 1990s and has been successfully implemented in various places across the USA, Canada and Europe –especially Finland– are developed around two postulates. The first is to provide housing and the second is to provide necessary support for health, well-being, and social integration. This initiative is aimed at underprivileged populations in general, but especially the homeless, those engaged in prostitution and undocumented immigrants, and the results are significant. Several other studies [60–66] also underscore the existence of a strong relationship between the lack of adequate housing and increased mortality among homeless individuals, which is three to four times higher than that of the general population. They also report a life expectancy of 42 to 52 years for homeless individuals, which is approximately 30 years lower than that of the general population [67].

However, despite the demonstrated beneficial outcomes of these interventions, none of the participant responses in this study indicated knowledge of any similar programs operating in Spain. Rather, they point to the acute absence of specific actions to facilitate accessibility of healthcare services to homeless individuals. The only known initiative was implemented in Barcelona from 2010 to 2014, based on an agreement between the Barcelona city government and the NGO *Médicos del Mundo*. This entity provided assistance to homeless individuals and other vulnerable populations from a mobile medical bus that circulated in the city and gave patients direct access to care, with no administrative or documentation pre-requisites. Though quite successful, the program was not renewed. Though, a few cities have similar collaboration agreements with NGOs to provide mobile medical care that makes it possible to attend to patients in situ, they are sporadic, irregular, and geographically limited. Currently, there are no specific services generally available to this population in Spain.

Along with continuity and political will, the success of these models requires the support of health departments and social services [68]. However, we found that social policies oriented to this group are completely inadequate and insufficient to mitigate the difficulties of access for homeless individuals to public health services in equal conditions to other users. This leaves them in a state of extreme fragility and double exclusion, in which they must cope with medical exclusion in addition to socioeconomic exclusion.

### Limitations and strengths of the study

This study collected the responses of the target population group, along with those of healthcare professionals –doctors, nurses, and social workers– and other professionals linked to public social services or NGOs. The aim was to achieve a more global assessment, compared to prior studies that only recorded responses from one of the implicated groups. The qualitative approach was optimal for analyzing the existence of inequities as well as their causes and consequences. It also served to formulate corresponding recommendations or intervention pathways for political decision-makers or healthcare professionals. However, these findings could be affected by specific research weaknesses. First, the research was done exclusively in the city of Barcelona and caution is advised regarding transferring the findings to other cities. Second, given the sampling procedures and non-compulsory participation of the individuals selected, in some cases the study may have been affected by issues derived from participant self-selection. Finally, the researchers based the study on services, entities and individuals who habitually work with the homeless, which may have biased the opinions of the work team regarding such services, or their assessment of the participant responses.

### Recommendations

From the results of this research, we can deduce that modifications are needed in the provision of public health services, if inequity of access and use by certain population groups is to be avoided. In policymaking, it would be necessary to alter facilities so that medical professionals can give adequate service to this population (showers, individual examination rooms, etc.) and streamline the procedure for accessing services, to avoid the bureaucratic gatekeeping role during initial contact. At the same time, a system for providing in situ medical attention could be implemented, such as a mobile clinic that goes round the city, making stops in places where the homeless population is denser. Along similar lines is the possibility of pertinent NGOs doing minor medical interventions on the street or at their own facilities. Finally, practitioners need more training and increased sensitivity to homeless individuals, so that the care they provide makes adequate use of public resources. This may also help prevent stigmas and attitudes from exacerbating the fragile social and medical situation of this population group.

### Unanswered questions and future research lines

During this study, some questions have remained unanswered. Though not the principal objective of the study, they might help to better understand the problem in its entirety.

Some professionals indicated that the conditions in their respective services do not facilitate giving adequate care to this population group. However, the information and data gathered in this study cannot verify that this is the case. As other studies indicate, inadequate care could be due to stigmatization of this group by medical professionals. Neither can we confirm that in situ interventions or easier access to services would increase homeless medical visits, as relevant information was not gathered in this study. Similarly, the study did not explore how bad experiences might affect the frequency of later visits to the doctor.

Some professionals implied that it is helpful when these individuals are accompanied to their doctor visits (social workers, volunteers, …) to facilitate more correct actions by the doctors, but such views cannot be verified from the information gathered in the study.

In future research, it would be interesting to expand the study to other cities, to determine if the same patterns occur for those implicated in the care process. It would also be interesting to replicate the study in countries that do not have the universal care provided in Spain, to contrast and compare how equity of access for this population is affected. Other issues that could be further explored include the relationship between conditioning factors in facilities, the attitudes of professionals and the attention given to these groups. This would consider the possibility that even if services are adapted, patient care might remain inadequate due to persistent attitudes. Finally, decision-makers and administrators could be included in future research, to compile responses from all those implicated in medical care processes.

## Conclusions

The results of this study demonstrate that homeless individuals encounter serious barriers to making standard use of public healthcare systems and thereby experience inequities of access. This situation primarily stems from the organization of these services but is also significantly influenced by the stigmas and attitudes of healthcare professionals regarding this population. While Spanish legislation establishes a system of universal access to healthcare services, this study verifies that in practice, certain groups encounter significant barriers to the access and use of healthcare services in equal conditions to the rest of the population. Public health systems have been designed for standard patients. To the degree that some population groups exhibit different characteristics or experience some type of stigma that differs from those of a standard patient, they will encounter certain barriers in accessing the services offered by the system.

## Data Availability

The datasets used or gathered for this study can be provided by the authors upon request in Spanish.
